# Double Trouble: Eales Disease in a Background of Paradoxical Embolism

**DOI:** 10.7759/cureus.44708

**Published:** 2023-09-05

**Authors:** David Horvath, Usama Aljameey, Elizabeth Douglas

**Affiliations:** 1 Ophthalmology, Lincoln Memorial University DeBusk College of Osteopathic Medicine, Harrogate, USA; 2 Family Medicine, Lincoln Memorial University DeBusk College of Osteopathic Medicine, Harrogate, USA

**Keywords:** retinal ischemia, retinal vasculitis, paradoxical embolism, patent foramen ovale, brao, brvo, eales disease

## Abstract

Eales disease is an idiopathic retinal vasculitis that mainly affects the periphery of the retina. The disease commonly manifests as peripheral retinal perivasculitis, peripheral retinal capillary nonperfusion, neovascularization, and recurrent vitreous hemorrhage. Here, we present the case of a 36-year-old male who was diagnosed with Eales disease after presenting with sudden onset flashes of light, reduced visual acuity, and a black spot in his left eye. Upon examination, his left eye exhibited a superior non-foveal branch retinal artery occlusion (BRAO) with a sludged blood column, an old extramacular branch retinal vein occlusion (BRVO) with hemorrhage, and vascular sheathing. Initial laboratory investigations, including antibody testing for causes of retinal ischemia and stroke workup, were negative. Later, the patient presented with a BRAO in the right eye and a cerebral infarction shortly thereafter, further complicating his clinical picture. A diagnosis of Eales disease was made based on the evolution of retinal findings showing peripheral non-perfusion, vascular sheathing, collateral formation, neovascularization with leakage, absence of additional BRAOs following repair of his patent foramen ovale, and lack of other explanatory conditions. The initiation of systemic corticosteroids resulted in the improvement and stabilization of his vision. This case highlights the challenges in diagnosing Eales disease, underscoring the importance of timely identification for the appropriate management and prevention of vision loss.

## Introduction

Eales disease is an idiopathic retinal periphlebitis, usually manifesting in the form of a branch retinal vein occlusion (BRVO), which commonly affects the peripheral retina leading to complications of neovascularization, retinal detachment, and vitreous hemorrhage [1]. The disease is uncommon in North America but prevalent in the Indian, Asian, and Middle East territories, where tuberculosis infection rates are high [2]. Diagnosis is by exclusion and based on the characteristic clinical picture, fluorescein angiographic findings, and evolution through the venous inflammation, ischemia, and hemorrhage stages [3,4]. Herein, we present the case of a 36-year-old male who presented with an unknown cause of one-month onset, intermittent unilateral vision loss.

## Case presentation

A 36-year-old Caucasian male was referred to a retina specialist by his optometrist for a left eye BRVO. He noticed flashes of light, reduced visual acuity, and a black spot in his left eye over the last day. He reported intermittent blurry vision in the left eye over the last month. The patient denied trauma and changes in his right eye. He reported an insignificant systemic past medical history except for stage 1 hypertension, mild hyperlipidemia, and idiopathic large joint pain. He denied any symptoms of dyspnea, fever, chest pain, and weight loss. He has never received the tuberculosis vaccine. Aside from myopia, his ocular history was unremarkable. Medications include chlorthalidone, atorvastatin, and lisinopril. His family history was noncontributory. 

**Figure 1:**
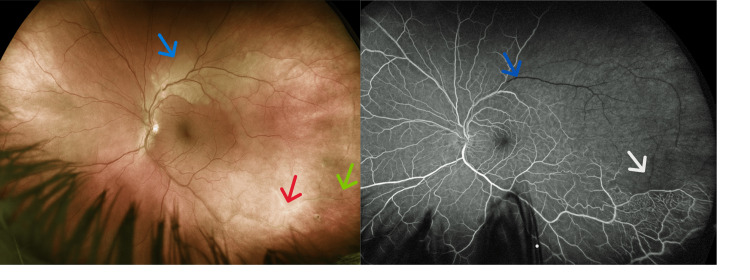
Optos ultra-widefield imaging (left) showing a BRAO (blue arrow), sclerotic vessels from an old inferior temporal BRVO (red arrow), and dot blot hemorrhages (green arrow). Vascular sheathing is far in the periphery and beyond the observable field of view. FA (right) showing filling defects in the superotemporal artery (blue arrow) and an old BRVO (white arrow).

Best corrected visual acuity was 20/20 in the right eye and 20/25 in the left eye. Confrontation to visual fields, extraocular motility, and pupils were normal. A slit-lamp examination revealed a normal anterior segment bilaterally with an intraocular pressure of 14 and 17 in the right and left eyes, respectively. The fundoscopic examination showed flat, sharp, and pink discs with a normal vitreous in both eyes. The right eye retina contained no abnormalities, while the left eye included a superior non-foveal BRAO with a sludged blood column, hemorrhage, sclerotic vessels from an old BRVO, vascular sheathing far in the periphery, and no neovascularization (Figure 1). Fluorescein angiography (FA) revealed filling defects in the superotemporal arcade and an old extramacular BRVO (Figure 1).

Initial stroke evaluation yielded negative results. Antinuclear antibodies, anti-phospholipid antibodies, anti-cardiolipin antibodies, lupus anticoagulant, antinuclear cytoplasmic antibodies, Lyme total antibody, fluorescent treponemal antibody absorption, factor V Leiden mutation, prothrombin gene mutation, and MRI of the brain were all negative. Erythrocyte sedimentation rate, complete blood count, QuantiFERON level, antithrombin III level, and protein C and S activity were all within the reference range. Hemoglobin A1c was 4.80. Homocysteine levels were slightly high at 15.5 mg/dL with repeat measurements showing normalization after prescribed folic acid 1 mg twice per day. The patient denied travel outside of the United States or exposure to tuberculosis. 

**Figure 2:**
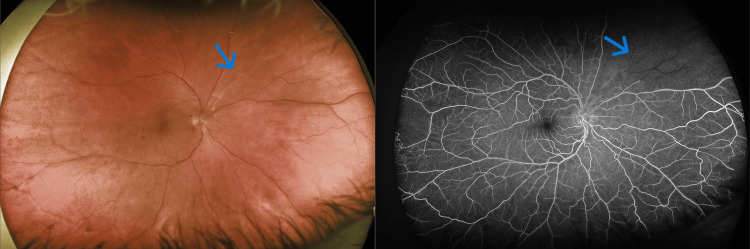
(Left) Nasal BRAO superonasally (blue arrow). (Right) FA showing a filling defect in the superior nasal field (blue arrow).

**Figure 3:**
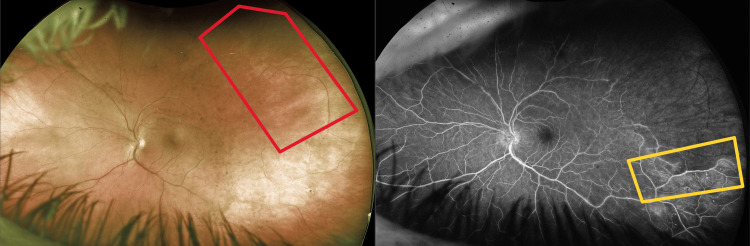
Follow-up Optos ultra-widefield imaging (left) demonstrating well-circumscribed areas of ischemia (red box) with FA (right) showing leakage of retinal vessels at the margin between the perfused and ischemic retina (yellow box).

At a later date, the patient presented urgently with complaints of blue flashing lights, floaters, and blurry vision in his right eye. A fundoscopic examination and Optos ultra-widefield imaging confirmed a new nasal BRAO in his right eye (Figure 2). Fundus imaging and FA of the left eye demonstrated well-circumscribed areas of ischemia with leakage of retinal vessels at the margin between the perfused and ischemic retina (Figure 3).

A transesophageal echocardiogram during the second stroke workup revealed a patent foramen ovale, potentially explaining the patient’s BRAOs. Shortly thereafter, the patient suffered a cerebral infarction, which prompted an immediate repair of the patent foramen ovale. Except for intermittent flashing lights, the patient’s vision remained stable over the next few months. 

**Figure 4:**
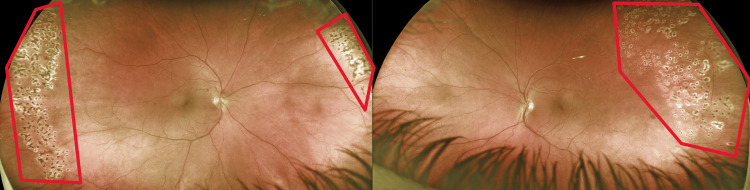
Panretinal photocoagulation burn scars in both eyes at various areas of ischemia (red outlines).

The degree of retinal vasculitis was largely consistent throughout follow-up exams and did not begin to show improvement until a prednisone 40 mg oral taper was prescribed. Periodically, the patient would return with complaints of photopsia and mildly blurred vision, both of which would resolve for weeks to months at a time after being given prednisone 40 mg oral taper. Over three settings, a panretinal photocoagulation laser was employed to prevent neovascularization and vitreous hemorrhage from leaky blood vessels (Figure 4). No further BRAOs were noted following the repair of the patent foramen ovale. 

Despite a negative antibody panel, the patient was referred to rheumatology services in search of a possible connection between his joint pain and retinal vasculitis. No definitive diagnosis was made, but methotrexate was offered to relieve eye and joint inflammation. Based on the medical history, the progression of retinal findings showing peripheral non-perfusion, segmental vascular sheathing, collateral formation, neovascularization with leakage, absence of subsequent BRAOs following repair of his patent foramen ovale, and lack of other explanatory conditions, the diagnosis of bilateral Eales disease was established.

## Discussion

The etiology of Eales disease remains unknown [1]. Observational studies show a purported relationship between nonviable *Mycobacterium tuberculosis* and a reactive type of inflammation in the retina [5]. These findings indirectly suggest a link between this disease and geographic regions located in the east. However, this explanation fails to account for the occurrence of this disease among Caucasian people in the United States, as observed in this case. The diagnosis of Eales disease is primarily based on clinical examination [3,4].

We present a case of Eales disease in a 36-year-old male with stage 1 hypertension, mild hyperlipidemia, and idiopathic large joint pain. BRVOs, which this patient experienced, may be noted on initial examination [5]. Up to 87% of patients eventually have bilateral involvement, similar to what transpired for this patient [3]. A fundoscopic examination revealed a superior non-foveal BRAO and an old extramacular BRVO, which explains the intermittent course of the disease and how the central part of the retina may already be affected on presentation [5]. 

On a return visit, the patient developed urgent complaints involving their right eye. Further examinations revealed a nasal BRAO in the right eye along with areas of ischemia and leakage of retinal vessels in the left eye. A transesophageal echocardiogram identified a patent foramen ovale that could account for both the BRAOs and the cerebral infarction that the patient subsequently experienced. The diagnosis of Eales disease was almost missed in this case due to the rarity of developing this disease in the absence of particular risk factors, such as exposure to *M. tuberculosis*. 

Eales disease tends to progress through the following stages: periphlebitis, capillary nonperfusion, neovascularization, and long-term complications of neovascularization, such as vitreous hemorrhage and retinal detachment [3,5]. Treatment is adjusted to the clinical stage. In cases where periphlebitis is evident, corticosteroids are warranted [3,5]. During non-perfusion and neovascularization, laser photocoagulation is recommended [3,5]. The combined use of corticosteroids and laser therapy may provide a synergistic effect [3,5]. Vitrectomy becomes necessary when complications of vitreous hemorrhage and tractional retinal detachments arise [3,5]. As mentioned here, the patient demonstrated signs of improvement in vision and inflammation periodically with systemic prednisone tapers. Laser photocoagulation was used to prevent neovascularization and vitreous hemorrhage.

## Conclusions

Eales disease is a rare and idiopathic retinal vasculitis that can cause vision loss due to complications, such as neovascularization, vitreous hemorrhage, and retinal detachment. It is more common in young males from certain geographic regions but can also affect other populations, especially with underlying risk factors. The diagnosis is based on clinical features, fluorescein angiography, and exclusion of other causes of retinal vasculitis with consideration for other pathophysiologic processes that may predict the underlying cause. Treatment options include anti-inflammatory agents, laser photocoagulation, and vitrectomy.

In this case report, we present a 36-year-old Caucasian male with bilateral Eales disease who developed paradoxical embolism due to a patent foramen ovale, leading to multiple episodes of branch retinal artery occlusions and cerebral infarction. This case highlights the importance of a thorough systemic evaluation and multidisciplinary management of patients with Eales disease. We hope this case will raise awareness of the unique way that Eales disease may showcase itself, leading to a timelier diagnosis and treatment.
